# Dose-dependent effects of capsaicin on intestinal morphology and microbiota composition in mice: Structural, immunohistochemical, and microbial insights

**DOI:** 10.14202/vetworld.2025.1703-1714

**Published:** 2025-06-26

**Authors:** Kai Li, Jianghai Xu, Siying Chen, Aifei Du, Shaohua Feng, Shibin Yuan, Bangyuan Wu

**Affiliations:** 1 College of Life Sciences, China West Normal University, Nanchong, 637000, Sichuan, China; 2 Yucai School Attached to Sichuan Chengdu No. 7 High School, Chengdu, 610021, Sichuan, China; 3 Key Laboratory of Southwest China Wildlife Resources Conservation, Ministry of Education, Nanchong, Sichuan, 637000, China; 4 Nanchong Key Laboratory of Wildlife Nutritional Ecology and Disease Prevention and Control, Nanchong, 637000, Sichuan, China

**Keywords:** capsaicin, dose-response, goblet cells, gut microbiota, intestinal barrier, mucosal injury, tight junction proteins

## Abstract

**Background and Aim:**

Capsaicin (CAP), the pungent component of chili peppers, possesses diverse bioactive properties, including antioxidant, anti-inflammatory, and antimicrobial effects. However, its impact on gastrointestinal integrity and microbial ecology remains dose-dependent and incompletely understood. This study aimed to investigate the effects of varying CAP doses on intestinal morphology, tight junction protein expression, goblet cell density, mucosal injury markers, and gut microbiota composition in mice.

**Materials and Methods:**

Seventy-five male Kunming mice were randomly assigned to five groups (n = 15/group): Normal control, vehicle control (dimethyl sulfoxide), low-dose CAP (5 mg/kg), medium-dose (15 mg/kg), and high-dose (20 mg/kg). Mice received oral gavage every other day for 14 days. Histological assessments (H&E and Alcian Blue-Periodic Acid–Schiff staining), enzyme-linked immunosorbent assays for diamine oxidase, fatty acid-binding protein 2, and plasma endotoxin as well as immunohistochemistry for ZO-1, Claudin-1, and Occludin, and 16S rRNA sequencing were employed to evaluate structural and microbial changes.

**Results:**

Low-dose CAP significantly enhanced villus height, reduced crypt depth, and elevated the villus-to-crypt ratio across all intestinal segments (p < 0.05). Tight junction protein expression and goblet cell counts were highest in the low-dose group, suggesting mucosal protection. In contrast, medium and high-dose CAP induced epithelial damage, villus atrophy, and downregulation of junctional proteins. Microbiota analysis revealed the suppression of Proteobacteria and the expansion of Firmicutes in the medium- and high-dose groups. All CAP doses stimulated microbial biosynthesis of cofactors, vitamins, and electron carriers, with enhanced alpha diversity at higher doses.

**Conclusion:**

CAP exhibits a biphasic effect on intestinal physiology. While low-dose administration supports mucosal integrity and promotes beneficial microbial functions, higher doses disrupt epithelial architecture and induce dysbiosis. These findings underscore the importance of dose consideration in CAP’s dietary and therapeutic applications, providing mechanistic insights into its gut-mediated effects.

## INTRODUCTION

Capsicum, introduced to China in the late 16^th^ century, is now extensively cultivated both domestically and globally. It holds substantial culinary and medicinal value, primarily attributed to capsaicin (CAP), its principal bioactive component, known for its gastroprotective and digestive-stimulating properties [1, 2]. CAP (trans-8-methyl-N-vanillyl-6-nonenamide) is an oxamide-containing alkaloid belonging to a family of structurally related compounds, including dihydrocapsaicin, nordihydrocapsaicin, and homocapsaicin. Notably, CAP and dihydrocapsaicin constitute over 90% of total capsaicinoids [3]. A growing body of evidence supports CAP’s therapeutic potential in reducing body weight [4, 5] and exerting antimicrobial, anti-inflammatory, analgesic, and antitumor effects, especially against lung and breast cancers [6–10]. An *in vivo* study by Panchal *et al*. [11] has further demonstrated its ability to modulate glucose and lipid metabolism, reinforcing its metabolic benefits. Beyond its systemic effects, CAP acts as a gastric tonic by enhancing gastrointestinal motility, stimulating gastric secretions and peristalsis, and improving appetite and digestion. It also alters intestinal microbiota composition and suppresses pathogenic microorganisms [12–14]. Additional pharmacological activities include anthelmintic, antipruritic, and sudorific properties, making CAP useful in preventing and managing animal diarrhea [15, 16]. Collectively, these attributes highlight CAP’s profound impact on gastrointestinal physiology, particularly on the intestinal epithelium. The intestinal tract serves dual roles as the primary site for nutrient absorption and as the largest lymphoid organ contributing to immune defense. It is critical for preventing pathogen and toxin invasion [17]. Key structural elements such as villi and the mucosal layer support its absorptive and protective functions. Villi are finger-like projections of the mucosa that increase the intestinal surface area, thereby enhancing nutrient absorption. They are covered by secretory epithelial cells that produce enzymes and mucus, maintaining barrier integrity [18]. The structural and functional integrity of villi is pivotal in preventing systemic inflammation [19] and is commonly evaluated using markers such as mucosal permeability, plasma endotoxin (ET), L-lactate, diamine oxidase (DAO), and intestinal fatty acid-binding protein 2 (FABP2) [16, 20]. The gut microbiota, composed of trillions of symbiotic microorganisms, further supports intestinal homeostasis. Dysbiosis can impair barrier function, leading to mucosal damage and immune dysregulation, which are associated with various gastrointestinal and systemic diseases. CAP, through activation of TRPV1 receptors, modulates gut motility, enhances barrier integrity, and regulates immune responses [21]. However, high doses may cause intestinal irritation and injury, whereas moderate intake appears to strengthen mucosal function and promote microbial balance. The gut microbiome evolves in concert with its host and plays a crucial role in nutrient metabolism, immune function, and intestinal motility [22, 23]. It contributes to carbohydrate fermentation and energy regulation [24] and supplies essential nutrients, including B and K vitamins, as well as short-chain fatty acids, which the host cannot synthesize independently [25]. The microbiota comprises beneficial, intermediate, and potentially pathogenic taxa that interact dynamically to maintain intestinal homeostasis and systemic health [26].

Although numerous studies have demonstrated the systemic pharmacological effects of CAP, including its anti-inflammatory, metabolic, and anticancer properties, its impact on gastrointestinal structure and function remains incompletely elucidated, particularly in the context of dose-dependent administration. Prior investigations have largely focused on the broad physiological and microbial effects of CAP, yet few have systematically evaluated the differential histological and immunohistochemical responses of the intestinal epithelium to graded CAP exposure. In addition, while it is recognized that CAP can modulate gut microbiota composition, the extent to which varying doses alter microbial diversity, taxonomic structure, and functional metabolic pathways has not been adequately explored. Most existing research lacks integrative analyses that concurrently assess intestinal morphology, tight junction integrity, goblet cell density, mucosal injury biomarkers, and microbiome functionality. Moreover, the dualistic nature of CAP– therapeutic at low concentrations and deleterious at high doses – warrants a nuanced investigation to delineate its threshold for toxicity versus benefit. These knowledge gaps limit the ability to formulate safe and effective dietary or therapeutic recommendations involving CAP, particularly for gastrointestinal health.

This study aimed to systematically investigate the dose-dependent effects of CAP on the structural and functional integrity of the intestinal tract and its associated microbiota in a murine model. Specifically, we sought to evaluate the impact of low (5 mg/kg), medium (15 mg/kg), and high (20 mg/kg) doses of CAP on intestinal morphology – measured by villus length, crypt depth (CD), and goblet cell density – as well as on the expression of tight junction proteins (ZO-1, Claudin-1, and Occludin), and levels of mucosal injury biomarkers (DAO, FABP2, and ET). In parallel, we assessed alterations in gut microbiota composition through 16S rRNA sequencing, alpha diversity indices, and predictive functional profiling of microbial metabolic pathways. By integrating histological, immunological, and microbiological approaches, this study aims to elucidate the threshold at which CAP transitions from a gut-protective to a gut-damaging agent. The findings are expected to inform the rational use of CAP in nutritional and therapeutic settings and to provide mechanistic insight into its gastrointestinal actions.

## MATERIALS AND METHODS

### Ethical approval

The study was approved by the Animal Care Committee of China West Normal University (2024LLSC0052). The environmental conditions and facilities for the animals met the national standards.

### Study period and location

The study was conducted from July 2023 to August 2024 at the Nanchong Key Laboratory of Wildlife Nutrition Ecology and Disease Control.

### Experimental grouping and dosing

Seventy-five 6-week-old male Kunming mice were sourced from the Laboratory Animal Centre of North Sichuan Medical College in Nanchong, Sichuan, China. The mice were randomly assigned to five groups, with 15 mice in each group with three replicates of five animals in each group, all weighing approximately 25 ± 5 g. Adaptive feeding was initiated for 7 days before the commencement of the study. All mice were subjected to gavage every other day as shown in Table 1, and they were provided with unrestricted access to food and water.

**Table 1:** Grouping and treatment of mice.

Group	Treatment	Sample size
NC group	0.15 mL of saline solution by gavage	n = 15
DMSO group	0.15 mL dimethyl sulfoxide solution by gavage	n = 15
The low-CAP group	5 mL/kg capsaicin dimethyl sulfoxide solution by gavage	n = 15
Medium-dose CAP group	15 mL/kg capsaicin dimethyl sulfoxide solution by gavage	n = 15
High-CAP group	20 mL/kg capsaicin dimethyl sulfoxide solution by gavage	n = 15

NC group=Natural control group, DMSO group=Dimethyl sulfoxide group, Low CAP group=Low capsaicin group, Medium CAP group=Medium capsaicin group, High CAP group=High capsaicin group

### Sample collection and tissue processing

Body weight was measured daily throughout the experimental period. On day 14, mice were anesthetized and blood samples were collected for serum preparation and stored at −20°C. The duodenum, jejunum, and ileum were aseptically excised and segmented into two portions. One portion was fixed in 4% paraformaldehyde (E672002, Sangon Biotech, China) at 4°C for 24 h for histological processing. Software analysis was performed to determine the effects of different CAP doses on the intestinal mucosa and villus length. The remaining tissues were snap-frozen in liquid nitrogen and stored at −80°C for subsequent analysis. The fecal trays were lined with sterile plastic wrap to prevent contamination. Fresh fecal samples were collected immediately post-defecation, transferred into sterile 10 mL tubes, and stored at −80°C for microbiota analysis.

### Histological analysis

Tissue fixation and washing were performed following the method of Feldman and Wolfe (2014). Samples were dehydrated in a graded ethanol series (75%, 85%, 95%, and 100%), cleared with xylene, and embedded in paraffin wax. Paraffin blocks were sectioned at 5 μm and stained with hematoxylin and eosin (H&E) (G1120, Solarbio, China). Histological observations were conducted under a light microscope to assess villus length and CD. The villus and crypt dimensions were quantified using the ViewPoint image analysis system, and the villus length-to-crypt depth (V/C) ratio was calculated.

### Goblet cell staining

Goblet cells were visualized using an Alcian Blue-Periodic Acid–Schiff (AB-PAS) staining kit (G1285, Solarbio Biotech, China). Tissue sections were prepared as described above. Sections were washed, treated with periodic acid, rinsed, and subsequently stained with Schiff’s reagent. The sections were washed with running water and finally stained with hematoxylin. The cells were differentiated with hydrochloric acid, washed with water, dehydrated with gradient alcohol, and transparentized with xylene. Finally, sections were mounted using neutral resin. Stained sections were examined and photographed using a DM4B microscope (Leica, Wetzlar, Germany), and goblet cells were quantified using Image Pro Plus 6.0 software. Images were captured under a 20× objective lens with a spatial calibration of 2.0 pixels/μm. Positive cells were identified by threshold segmentation based on HSV parameters: Hue 250°–310°, saturation >30%, and value >50%.

### Enzyme-linked immunosorbent assays (ELISA) for intestinal markers

ELISA was conducted to measure DAO (ml002199), FABP2 (ml037218), and ET (ml002005) levels purchased from Shanghai Enzyme Linked Biotechnology (Shanghai, China). All assays were performed according to the manufacturer’s instructions. In summary, diluted standards were mixed with samples in enzyme plates. Samples were incubated with labeled detection antibodies. After washing the plates 3 times with PBS (No. E607008, Sangon Biotech, China), free HRP was introduced and incubated at 37°C for 30 min. Following another wash cycle, substrates A and B were added in the dark and incubated. Finally, the enzyme plate was removed, and a termination solution was applied. Absorbance was measured at 450 nm using a microplate reader (Epoch, BioTek, Agilent, USA), and data were analyzed.

### Immunohistochemistry

Intestinal tissues were processed using standard paraffin-embedding procedures and dehydrated with graded ethanol. Endogenous peroxidase activity was blocked using 3% hydrogen peroxide. The sections were washed with distilled water and rinsed with phosphate-buffered saline (PBS). We subsequently incubated the cells at room temperature with antibodies against ZO-1 (ml037693), Claudin-1 (ml037797), and Occludin (ml063481), all obtained from Shanghai Enzyme-Linked Biotechnology in Shanghai, China. Endogenous enzymes were inhibited by incubation with 3% H_2_O_2_, followed by boiling in 0.01 M sodium citrate buffer for 20 min, and then cooling at room temperature. Non-specific binding was blocked by incubating sections in 5% BSA. After the incubation period, the sections were washed 3 times with PBS. The slides were then incubated with a universal HRP-conjugated secondary antibody (ml087269) at 37°C. The sections were then washed twice with PBS and stained with DAB chromogen for 4 min, after which they were rinsed with water. To enhance the staining differentiation, a solution of 1% hydrochloric acid in ethanol was applied, followed by staining with a saturated lithium carbonate solution until a blue hue developed. The sections were then rinsed under running water. Subsequently, the samples were dehydrated using a gradient of ethanol and cleared with xylene. Under a microscope, positive immunoreactivity for ZO-1, Claudin-1, and Occludin was visualized by brown-yellow staining. Protein expression was semi-quantitatively analyzed using ImageJ (v1.53k) software (https://imagej.net/ij/) by measuring grayscale intensity values. Raw TIFF images were background-subtracted (rolling ball, radius=50 pixels). The integrated density of each band was normalized to β-actin. Statistical significance was determined by one-way analysis of variance with Tukey’s *post hoc* test.

### DNA extraction and microbial sequencing

#### Paired-end sequencing of colonic DNA

Total genomic DNA was extracted from fecal samples, and a cDNA library was constructed using mRNA hybridization with specific aptamers. The library was amplified by PCR and prepared for sequencing using specific primers targeting the 16S rRNA gene (F: ACTCCTACGGGAGGCAGCA, R: GGACTACHVGGGTWTCTAAT). Sequencing was performed for taxonomic identification and quantification of microbial communities using bipartite 16S_V3V4a regions. Sequencing was conducted on an Illumina MiSeq platform with paired-end reads of 2 × 150 bp. Quality filtering and data processing were performed using DADA2, VSEARCH, FrameBot, and the pheatmap R package.

### Bioinformatics and functional prediction

Amplicon sequence variant (ASV) and opera- tional taxonomic unit (OTU) clustering was performed using QIIME2 version 2020.11 (//qiime2.org/), DADA2 version 1.16 (//benjjneb.github.io/dada2/tutorial.html), and R packages 4.4.2 (//cran.r-project.org/), including VennDiagram, metagenomeSeq, and vegan. ASV analysis in QIIME2 (2020.11) and DADA2 (1.16) involved importing raw sequencing data, denoising with DADA2, clustering ASVs (in DADA2), assigning taxonomy, visualizing results, and exporting the ASV table for further analysis. Similarly, OTU analysis in R, utilizing packages such as VennDiagram, metagenomeSeq, and vegan, included importing raw OTU data, visualizing OTU distribution and overlap with Venn diagrams, performing data normalization and statistical analysis with metagenomeSeq, calculating diversity indices and assessing community structure with vegan, and exporting the OTU table for subsequent analysis, ASVs/OTUs were ranked by abundance within each sample or group, from highest to lowest. The values are arranged from largest to smallest. The relative abundance was plotted on the y-axis using polylines to visualize the ASV/OTU distribution patterns, thereby clearly displaying the abundance distribution across different samples. Log2 transformations were applied to abundance values to normalize data distribution (alternatively, a Log10 transformation, percentage transformation, or no transformation may also be selected) for the ordinate. Using R software, scripts were written to generate the abundance grade curve for each sample or population. Abundance-rank curves were generated to assess the distribution of dominant versus rare ASVs/OTUs across samples. Taxonomic assignment was performed using the Greengenes, KEGG, MetaCyc, and COG reference databases. Greengenes, KEGG, MetaCyc, and COG databases are used for taxonomic assignment and functional annotation by comparing ASVs against reference sequences or groups. Greengenes is primarily used for taxonomic classification of 16S rRNA gene sequences, KEGG is used for annotating functional genes and metabolic pathways, MetaCyc provides detailed metabolic pathways for organisms, and COG is used for identifying orthologous protein clusters and their associated functions across species. The results were analyzed using the Aromaticity Index and Simpson’s Diversity. The functional predictions of the microbial communities were conducted following the standard metagenomics tutorial protocol. Normalized pathway abundance tables were used to compare functional group distributions among the experimental groups. The FitFeatureModel function was applied to fit pathway distributions using a zero-inflated lognormal model. The statistical significance of the intergroup differences was evaluated based on the model-fitting outputs.

### Statistical analysis

Statistical analyses were performed using a one-way analysis of variance in SPSS (version 26.0, IBM SPSS, NY, USA), while graphs were generated using Origin 2022. Results are expressed as mean ± standard deviation. Statistical significance was defined as follows: p < 0.01, highly significant; p < 0.05, significant; and p > 0.05, not significant.

## RESULTS

### Morphological changes in the small intestine

**Supplementary Figure 1:**
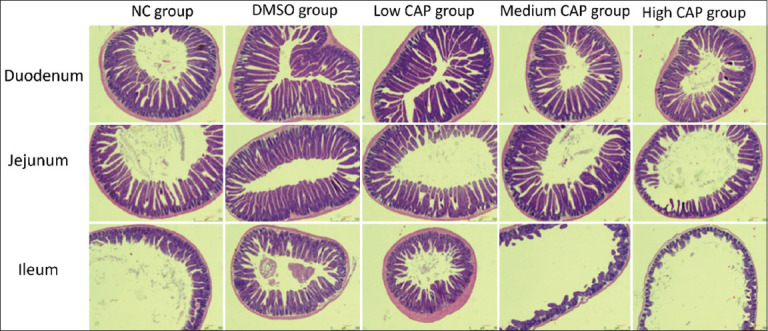
Structural changes in the duodenum, jejunum, and ileum (H&E staining, 100×).

In the normal control (NC) and dimethyl sulfoxide (DMSO) control groups, the duodenum, jejunum, and ileum maintained intact structural integrity, with no observable pathological lesions. In contrast, CAP administration resulted in dose-dependent histological alterations in villus architecture and epithelial integrity. The low-CAP group exhibited elongated duodenal villi, with no significant morphological changes observed in the jejunum or ileum. In the medium-CAP group, mild villus injury was noted in the duodenum and jejunum, whereas the ileum displayed extensive epithelial necrosis and structural damage. The high-CAP group exhibited the most severe histopathological changes, including villus shortening and crypt deepening in the duodenum, although partial structural preservation of duodenal villi was still evident. In contrast, the jejunum and especially the ileum showed marked villus atrophy and widespread epithelial necrosis. These observations confirm a dose-dependent relationship between CAP administration and small intestinal injury. Low doses potentially enhance duodenal absorption, whereas medium and high doses cause significant structural and cellular damage, most severely in the ileum, as shown in Supplementary Figure 1.

### Villus length, crypt depth, and V/C Ratio

By analyzing and comparing the lengths of villi and the depths of crypts in the duodenum, jejunum, and ileum across different concentration gradients, the villus length to crypt depth (V/C) values were calculated (Table 2). The results indicated that villus length in the duodenum, jejunum, and ileum was significantly increased in the low-CAP group (p < 0.05) relative to the NC and DMSO control groups. Conversely, villus length was significantly reduced in the medium and high CAP groups compared with the control group (p < 0.05). Notably, the longest villus length was observed in the low-CAP group, whereas the medium- and high-CAP groups exhibited the shortest villus lengths.

**Table 2:** Changes in the chorionic VH, CD, and VH/CD ratio.

Group	Duodenum	Jejunum	Ileum
Villus length (μm)			
NC group	11.81^a^ ± 558.82	12.10^a^ ± 479.23	9.56^a^ ± 453.26
DMSO group	9.89^a^ ± 543.36	10.23^a^ ± 465.65	14.85^a^ ± 467.85
The low-CAP group	10.87^b^ ± 590.33	13.56^b^ ± 512.49	13.27^b^ ± 456.59
Medium-dose CAP group	9.56^c^ ± 479.84	18.79^c^ ± 432.24	8.35^c^ ± 459.26
High-CAP group	8.98^d^ ± 457.25	9.56^d^ ± 407.99	7.89^d^ ± 443.29
CD (μm)			
NC group	5.65^a^ ± 135.30	7.45^a^ ± 123.19	4.57^a^ ± 151.65
DMSO group	4.21^a^ ± 119.15	4.87^a^ ± 130.43	6.52^a^ ± 155.85
The low-CAP group	3.24^a^ ± 102.67	5.63^b^ ± 119.18	9.75^a^ ± 154.25
Medium-dose CAP group	4.56^b^ ± 120.26	3.54^a^ ± 134.65	4.12^b^ ± 160.81
High-CAP group	7.86^b^ ± 151.91	3.21^a^ ± 132.46	3.15^b^ ± 167.54
V/C			
NC group	0.45^a^ ± 4.13	0.89^a^ ± 3.89	0.56^a^ ± 2.87
DMSO group	0.78^a^ ± 4.56	0.45^a^ ± 3.57	0.41^a^ ± 3.02
The low-CAP group	0.24^b^ ± 5.75	0.75^b^ ± 4.30	0.57^a^ ± 2.96
Medium-dose CAP group	0.35^c^ ± 3.99	0.23^c^ ± 3.21	0.21^b^ ± 2.84
High-CAP group	0.21^d^ ± 3.01	0.45^c^ ± 3.08	0.35^b^ ± 2.65

The difference between the same lowercase letters in the groups was not statistically significant (p > 0.05). There are no statistically significant differences between upper case letters in the group, and the difference between different letters was statistically significant (p < 0.05). The same follows. NC=Natural control, DMSO=Dimethyl sulfoxide, CAP=Capsaicin, VH=Villus height, CD=Crypt depth , V/C=Villus-to-crypt ^a-d^Letters in the same row indicate no significant difference (p > 0.05), while different letters indicate a significant difference (p < 0.05).

There was no significant difference in CD between the duodenum and ileum among the NC, DMSO, and low CAP groups (p > 0.05). However, notable differences emerged between the medium CAP group and the high CAP group when compared with the other groups, with an upward trend (p > 0.05). The lowest CD in the jejunum was recorded in the low and medium CAP groups, which was significantly different from the other groups (p < 0.05). Moreover, CD in the duodenum and jejunum was significantly lower in the low-CAP group (p < 0.05).

The V/C ratios in the duodenum and jejunum were significantly higher in the low and medium CAP groups than in the control groups (p < 0.05). In addition, there was a significant difference in the high CAP group across the three intestinal segments compared with the NC and DMSO groups, while the high CAP group exhibited a significantly reduced V/C ratio across all segments (p < 0.05). Notably, the V/C value was highest in the low-CAP group across all intestinal segments.

### Goblet cell changes

No significant differences in goblet cell counts were observed between the NC and DMSO control groups in the duodenum, jejunum, and ileum, indicating that DMSO as a solvent had no effect on the small intestine. The administration of low-dose CAP did not result in a significant change in goblet cell numbers. In contrast, medium and high-dose CAP significantly reduced goblet cell numbers in all small intestinal segments, with the greatest reduction observed in the high-CAP group. A proximal-to-distal gradient in goblet cell abundance was observed, with higher counts in the ileum than in the duodenum. Furthermore, CAP’s impact exhibited a dose-dependent reduction across all intestinal segments, with the ileum consistently exhibiting the highest goblet cell densities among all segments. Overall, CAP altered goblet cell density in a dose-dependent manner, with medium and high concentrations significantly suppressing goblet cell populations. The analysis revealed significant differences in the duodenum, jejunum, and ileum between the medium CAP, high CAP, NC, DMSO, and low CAP groups (p < 0.05). However, no significant differences were observed within each intestinal segment among the NC, DMSO, and low CAP groups (p > 0.05). Notable differences were observed in the duodenum, jejunum, and ileum between the NC, DMSO, and low CAP groups (p < 0.05). Significant differences were also identified between the medium CAP and DMSO groups, as well as between the medium CAP and high CAP groups, in both the jejunum and ileum (p < 0.05). In the low CAP group, which encompasses the duodenum, jejunum, and ileum, the number of goblet cells was higher than that in the NC group (Table 3). These findings suggest that low-dose CAP may have beneficial effects on the small intestine.

**Table 3:** Changes in the number of goblet cells.

Group	Duodenum	Jejunum	Ileum
NC group	103.89 ± 14.57^a^	119.56 ± 7.38^d^	147.48 ± 9.76^g^
DMSO group	102.85 ± 16.98^a^	113.76 ± 8.76^d^	143.65 ± 11.05^g^
The low-CAP group	109.76 ± 16.48^a^	124.59 ± 8.69^d^	147.94 ± 16.75^g^
Medium-dose CAP group	60.57 ± 8.64^b^	66.98 ± 7.85^e^	90.87 ± 6.79h
High-CAP group	54.09 ± 7.38^c^	59.38 ± 8.27^f^	86.85 ± 6.38i

Abbreviations: NC=Natural control, DMSO=Dimethyl sulfoxide, CAP=Capsaicin ^a-i^Letters in the same row indicate no significant difference (p > 0.05), while different letters indicate a significant difference (p < 0.05).

### Mucosal injury markers (DAO, ET, FABP2)

**Figure 1:**
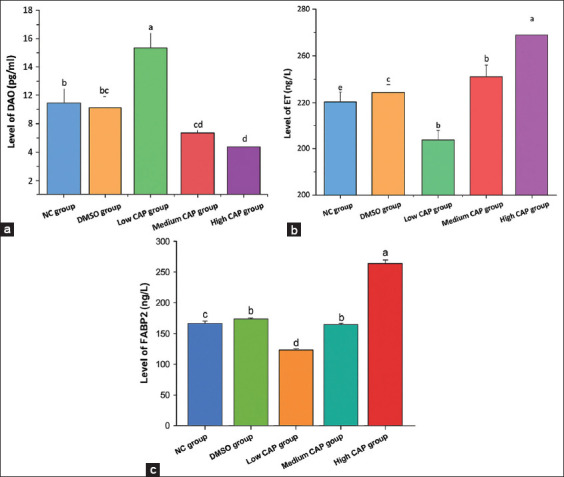
Changes of intestinal mucosal injury. (a) DAO content, (b) ET content, (c) FABP2 content. DAO=Diamine oxidase, ET=Endotoxin, FABP2=Fatty acid-binding protein 2.

**Figure 2:**
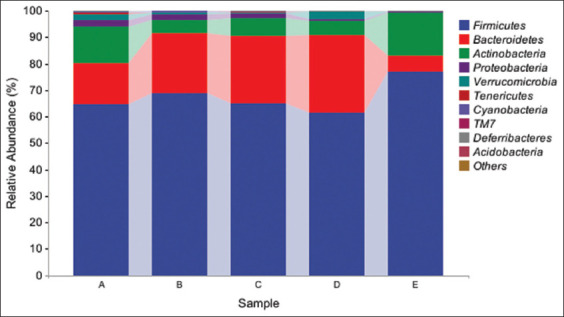
Species richness of groups at the gate level (intra-group means). A=Normal control group, B=Low CAP group, C=Medium CAP group, D=High CAP group, E=DMSO group, CAP=Capsaicin.

As shown in Figure 1, DAO levels were significantly elevated in the low CAP group relative to the NC group (p < 0.05). Conversely, DAO levels were markedly reduced in the medium and high CAP groups (p > 0.05). No significant differences were observed between the NC and DMSO groups (p > 0.05). As shown in Figures 2B and C, both ET and FABP2 levels exhibited an initial decline followed by a dose-dependent increase following CAP ingestion. Notably, ET and FABP2 concentrations were significantly elevated in the medium and high CAP groups compared with the NC controls. ET and FABP2 levels were significantly lower in the low CAP group than in the NC group.

### Tight junction protein expression

**Figure 3:**
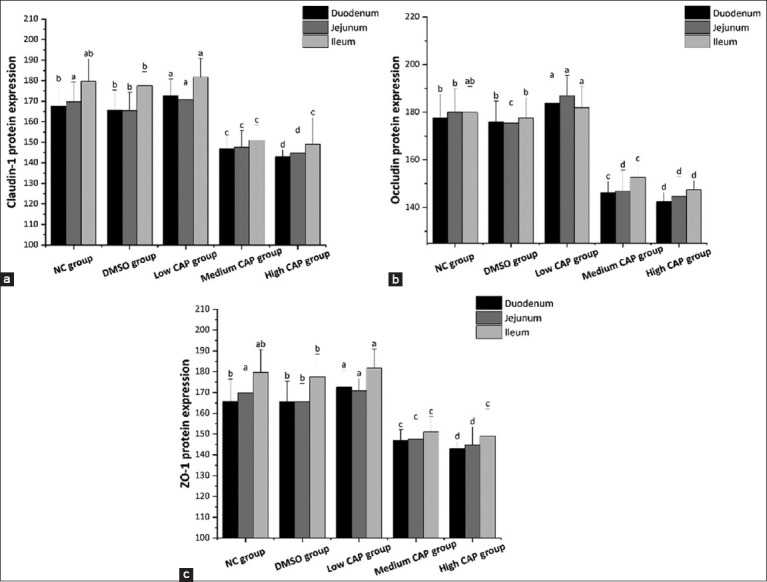
Expression of junction ZO-1, Claudin-1, and Occludin protein. (a) Claudin-1 protein expression, (b) Occludin protein expression, (c) ZO-1 protein expression.

As can be observed in Figures 3A-C, expression levels of ZO-1, Claudin-1, and Occludin declined significantly in a dose-dependent manner with increasing CAP administration (p < 0.05), with the highest expression observed in the low CAP group (p < 0.05). Expression levels in the medium and high CAP groups were significantly reduced compared with NC and DMSO controls (p < 0.05). The ileal expression of ZO-1, Claudin-1, and Occludin proteins was significantly lower in the NC and DMSO groups. In the ileum, Claudin-1 protein, Occludin protein, and ZO-1 protein changed significantly (p < 0.05), although the overall trend was not statistically significant in all segments.

### Microbiota composition (species analysis and diversity)

After comparing the compositional analysis of species, as shown in Figure 2, the results indicate that Firmicutes were predominant across all groups, with the highest relative abundance observed in the high CAP group, where Firmicutes accounted for the majority of detected microbial taxa. The dominant position of Firmicutes in these treatment groups suggests that CAP intake, particularly at high CAP doses, significantly promotes the proliferation of Firmicutes. In contrast, Proteobacterial abundance was markedly suppressed in groups exposed to CAP, particularly at medium and high doses. The reduction in the purple section in the medium CAP group and high CAP groups compared with the NC and DMSO groups indicates an inhibitory effect. The inhibitory effect of CAP on Proteobacteria was dose-dependent, intensifying with increasing concentrations.

### Alpha diversity

**Figure 4:**
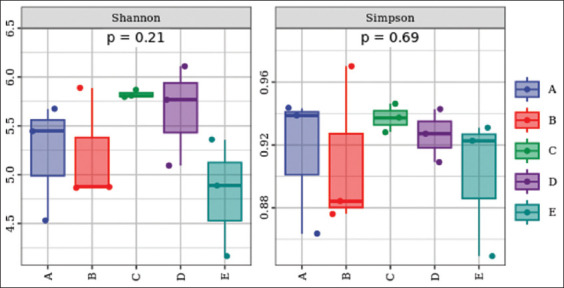
Alpha diversity box plot. A=Normal control group, B=Low CAP group, C=Medium-capacity CAP group, D=High CAP group, E=Dimethyl sulfoxide group, CAP=Capsaicin.

After comparing the species diversity of the community, the results are shown in Figure 4. In the medium and high CAP groups, the Aromaticity Index increased, while the Simpson’s Diversity Index decreased, relative to the NC group. No significant changes were found in the aroma index and Simpson’s index, indicating that the low CAP group had little or no effect on the diversity of intestinal microbial species.

### Functional potential projections

**Figure 5:**
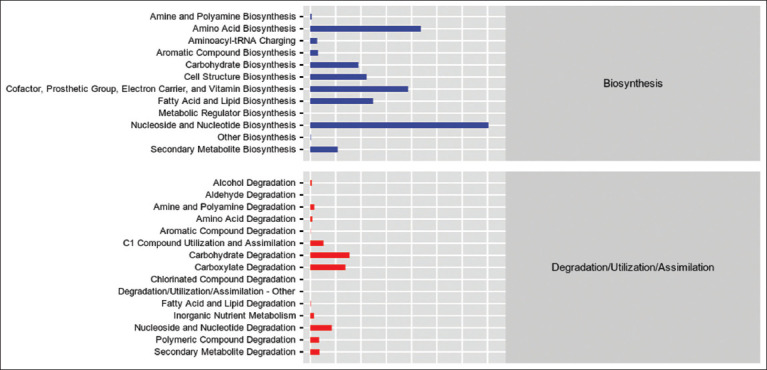
Metabolic pathway statistics overall secondary functional classes statistical map.

This study focused on two key functional categories of microbial metabolism: Biosynthesis, biodegradation, and utilization. The mean values for these functions were derived for each group and are presented in the secondary functional group statistics (Figure 5). Amino acid biosynthesis, along with cofactor, electron carrier, and vitamin biosynthesis, was most pronounced in the low CAP group. In contrast, nucleoside and nucleotide biosynthesis were more prominent in the NC and DMSO groups. All groups exhibited high metabolic activity in carbohydrate and carboxylate degradation pathways. Nucleoside and nucleotide degradation activity was significantly reduced in the high CAP and DMSO groups compared with the other treatments.

## DISCUSSION

### CAP’s dose-dependent effects on intestinal morphology

This study demonstrated that CAP significantly influences both intestinal architecture and gut microbiota composition in a dose-dependent manner, with medium and high doses causing substantial damage to the intestines. Capsicum is commonly used as both a medicinal and culinary agent. CAP is the principal and most bioactive compound in capsicum [27], representing not only the most abundant but also the most pharmacologically potent constituent. Chronic oral administration of CAP has been shown to modulate host physiological responses, intestinal morphology, and the dynamics of the microbial community. In the present study, intestinal morphology was evaluated using histological sectioning (H&E staining), while the expression of epithelial cell junction proteins—ZO-1, Claudin-1, and Occludin—was assessed using immunohistochemistry. Goblet cell numbers were quantified through AB-PAS staining to assess mucosal integrity and potential damage induced by CAP.

### Histological and barrier integrity alterations

The findings revealed that higher doses of CAP-induced significant intestinal damage, potentially through the upregulation of inflammatory mediators [28], leading to mucosal thinning and villus degradation. The high CAP group exhibited the most pronounced intestinal injury, followed by the medium-CAP group. Both showed marked reductions in villus length and expression of tight junction proteins compared to the NC and DMSO controls. In contrast, the low-CAP group exhibited the longest villi, surpassing even the NC group, suggesting a promotive effect on villus growth and tight junction integrity.

CD was significantly increased in the medium- and high-CAP groups, while it was lowest in the low-CAP group. The villus-to-crypt (V/C) ratio was elevated in both the low- and medium-dose groups but significantly reduced in the high-CAP group. The highest V/C ratio was observed in the low-CAP group. These results highlight the protective and trophic effects of low-dose CAP, as well as the pathological changes induced by higher doses.

### Goblet cell dynamics and mucosal protection

The elevated goblet cell count in the low-CAP group may reflect a compensatory response to subtle epithelial disturbances, triggering inflammatory signaling that increases mucus production [29]. In contrast, goblet cell numbers were significantly reduced in both the medium- and high-CAP groups, likely due to damage to the mucosal barrier. As CAP concentration increased, epithelial cell density and goblet cell numbers decreased. Since these cells are crucial for maintaining the mucosal barrier [30], their loss impairs intestinal defense mechanisms. Goblet cells secrete connexins that support tight junction formation; a reduction in these cells results in decreased connexin levels and compromised epithelial integrity [31, 32].

### Impact on tight junction proteins and permeability

**Figure 6:**
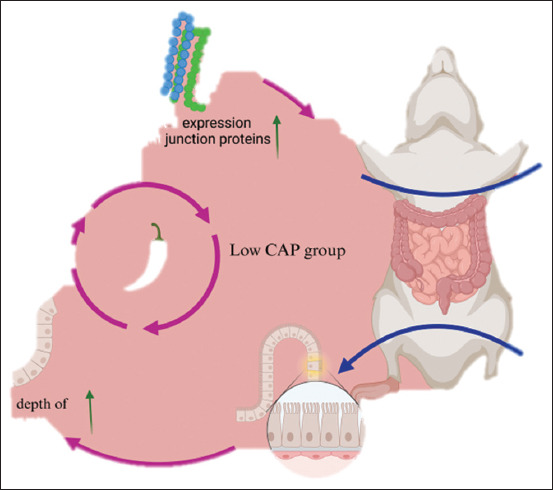
Effects of capsaicin on intestinal structure and microbiota.

The present study demonstrated that Occludin, Claudin-1, ZO-1, and DAO levels decreased with increasing CAP concentration, while low-dose CAP enhanced their expression compared to the NC and DMSO controls. High-dose CAP disrupted epithelial tight junctions and increased permeability [33, 34], leading to reduced villus height (VH), weakened barrier function, and impaired nutrient absorption [35]. Low-dose CAP, conversely, may enhance digestive and absorptive capacity by maintaining epithelial integrity**,** as shown in Figure 6.

### Shifts in microbiota composition

The gut microbiota is a complex ecosystem with essential symbiotic roles [36]. In the NC and DMSO groups, *Thickettia* *Anabaena*, and *Actinobacteria* were dominant, consistent with prior murine studies [37]. CAP treatment suppressed *Proteobacteria* while promoting *Firmicutes*, especially in the high-CAP group. Interestingly, CAP increased *Anopheles* abundance across all doses, possibly due to its counteractive effects on DMSO toxicity or direct promotion of specific microbial taxa [38].

In addition, *Actinobacteria* abundance declined in all CAP-treated groups. *Aspergillus spp*., which includes several pathogenic species (e.g., *Escherichia coli*, *Helicobacter pylori*), was also significantly reduced by high-dose CAP [39, 40], suggesting its antimicrobial potential.

### Diversity and functional capacity of the microbiota

Alpha diversity analyses revealed increased diversity in the medium- and high-CAP groups, while no changes were observed in the low-CAP group. Functional predictions demonstrated heightened nucleoside and nucleotide biosynthesis across all groups, likely reflect-ing active microbial replication. CAP also enhanced the microbial biosynthesis of cofactors, electron carriers, and vitamins – functions attributed to beneficial taxa such as *Bacillus* spp., which produce vitamin K2 [41, 42].

### Metabolic function and enzyme suppression

The low-CAP group showed the strongest microbial amino acid biosynthesis activity. In contrast, nucleoside and nucleotide catabolism were significantly suppressed in the high-CAP group, suggesting enzyme inhibition [43, 44]. CAP’s effect on microbial metabolism appears to be dose-dependent, with high concentrations potentially inhibiting nucleotidase activity and impairing nucleotide turnover.

## CONCLUSION

This study comprehensively demonstrated that CAP exerts dose-dependent effects on intestinal morphology and gut microbiota composition in mice. Low-dose CAP (5 mg/kg) was found to enhance gastrointestinal health by increasing VH, reducing CD, elevating the villus-to-crypt ratio, and upregulating tight junction proteins (ZO-1, Claudin-1, Occludin). It also preserved goblet cell density and improved mucosal barrier integrity. In contrast, medium (15 mg/kg) and high (20 mg/kg) doses induced significant histological damage, including epithelial necrosis, villus atrophy, and a reduction in goblet cell numbers. These higher doses also downregulated barrier-associated proteins and increased biomarkers of mucosal injury (DAO, ET, FABP2).

Microbiota analyses revealed that CAP altered gut microbial composition in a concentration-dependent manner. While low doses modestly modulated microbial taxa, medium and high doses notably suppressed Proteobacteria and promoted the proliferation of Firmicutes. Functional predictions further indicated enhanced microbial biosynthesis of vitamins, cofactors, and amino acids, particularly in the low-CAP group. High-dose CAP, however, was associated with suppressed nucleotide degradation functions and reduced alpha diversity indices, suggesting potential dysbiosis.

These findings offer critical insights for developing CAP-based dietary or therapeutic interventions. Low-dose CAP may be safely incorporated into functional foods or nutraceuticals to support intestinal health, enhance epithelial integrity, and modulate beneficial gut microbiota.

The study employed an integrative approach combining histological, immunohistochemical, bio chemical, and microbiome analyses. The use of clearly stratified CAP doses and rigorous assessment of both host and microbial parameters is a major strength.

The study was limited to a 14-day administration period and male mice, which may not fully capture long-term or sex-specific effects. Functional validation of predicted microbial pathways and host inflammatory markers was not conducted.

Future investigations should assess the chronic effects of CAP, explore sex-based differences, and evaluate host–microbiota interactions using metatranscriptomics or metabolomics. Studies involving disease models may further clarify CAP’s therapeutic potential in intestinal disorders.

In summary, this study provides evidence that low-dose CAP supports intestinal homeostasis, while excessive intake compromises epithelial integrity and microbial balance. These results underscore the importance of dose consideration in CAP application and pave the way for its rational use in gut-targeted health interventions.

## AUTHORS’ CONTRIBUTIONS

JX and AD: Conceptualization. SF: Data curation, resources, and validation. KL: Formal analysis, supervision, and writing – review and editing. BW: Project administration. AD: Investigation and writing – original draft. KL and JX: Methodology. SF, JX, SC, and SY: Software, statistical analysis, and visualization. All authors have read, reviewed, and approved the final manuscript.
